# Estimation of outbreak severity and transmissibility: Influenza A(H1N1)pdm09 in households

**DOI:** 10.1186/1741-7015-10-117

**Published:** 2012-10-09

**Authors:** Thomas House, Nadia Inglis, Joshua V Ross, Fay Wilson, Shakeel Suleman, Obaghe Edeghere, Gillian Smith, Babatunde Olowokure, Matt J Keeling

**Affiliations:** 1 Mathematics Institute, University of Warwick, Coventry, CV4 7AL, UK; 2 Health Protection Agency West Midlands, 5 St Phillips Place, Birmingham, B3 2PW, UK; 3 Operations Research and Statistics Group, School of Mathematical Sciences, University of Adelaide, Adelaide, SA 5005, Australia; 4 BADGER Group, Badger House, 121 Glover Street, Birmingham, B9 4EY, UK; 5 School of Life Sciences, University of Warwick, Coventry, CV4 7AL, UK

**Keywords:** Influenza A(H1N1)pdm09, Household, Case ascertainment, Markov Chain Monte Carlo, Transmission dynamics

## Abstract

**Background:**

When an outbreak of a novel pathogen occurs, some of the most pressing questions from a public-health point of view relate to its transmissibility, and the probabilities of different clinical outcomes following infection, to allow an informed response. Estimates of these quantities are often based on household data due to the high potential for transmission in this setting, but typically a rich spectrum of individual-level outcomes (from uninfected to serious illness) are simplified to binary data (infected or not). We address the added benefit from retaining the heterogeneous outcome information in the case of the 2009-10 influenza pandemic, which posed particular problems for estimation of key epidemiological characteristics due to its relatively mild nature and hence low case ascertainment rates.

**Methods:**

We use mathematical models of within-household transmission and case ascertainment, together with Bayesian statistics to estimate transmission probabilities stratified by household size, the variability of infectiousness of cases, and a set of probabilities describing case ascertainment. This novel approach was applied to data we collected from the early "containment phase" stage of the epidemic in Birmingham, England. We also conducted a comprehensive review of studies of household transmission of influenza A(H1N1)pdm09.

**Results:**

We find large variability in the published estimates of within-household transmissibility of influenza A(H1N1)pdm09 in both model-based studies and those reporting secondary attack rates, finding that these estimates are very sensitive to how an infected case is defined. In particular, we find that reliance on laboratory confirmation alone underestimates the true number of cases, while utilising the heterogeneous range of outcomes (based on case definitions) for household infections allows a far more comprehensive pattern of transmission to be elucidated.

**Conclusions:**

Differences in household sizes and how cases are defined could account for an appreciable proportion of the reported variability of within-household transmissibility of influenza A(H1N1)pdm09. Retaining and statistically analysing the full spectrum of individual-level outcomes (based on case definitions) rather than taking a potentially arbitrary threshold for infection, provides much-needed additional information. In a future pandemic, our approach could be used as a real-time analysis tool to infer the true number of cases, within-household transmission rates and levels of case ascertainment.

## Introduction

Emerging infectious diseases remain an ongoing and serious threat to human health. Determining the appropriate and measured response to any new threat is often guided by mathematical models, which critically depend on good estimates of key epidemiological parameters, such as transmission rates, case ascertainment and case severity. For respiratory pathogens such as influenza, the potential for a global pandemic is always present, however the early estimation of how virulent and transmissible a given organism may be remains extremely difficult. This is primarily because mild cases do not typically present themselves to the public health system, and so there is always the possibility that severe cases will be considered more typical than they actually are. The UK Department of Health currently emphasises the need to ascertain severity of a novel pandemic as soon as possible [1]. Uncertainty regarding the severity of the recent 2009 H1N1 pandemic, which was relatively mild in most cases compared to previous pandemics such as that in 1918-19, was a key problem for early efforts to estimate the epidemiological quantities necessary to inform public health policy [2,3].

Household data has formed a key part of efforts to estimate quantities relevant to the transmission dynamics of pandemic influenza [4,5]. The household is a natural unit for collection of epidemiological data for three main reasons. First, by definition members of a household are co-located and so are readily studied at the same time. Secondly, the close contacts between household members often lead to strong within-household transmission that provides rich information for statistical outbreak analysis. Finally, many interventions such as antiviral prophylaxis, treatment and isolation advice are often considered for targeting at the household level [6,7]. Households therefore form epidemiologically important units that are convenient to sample.

In this study, we use data on a large number of households (424) in Birmingham (England's second city and an early hotspot of the epidemic) affected during the first seven weeks of the 2009 H1N1 pandemic, to estimate within-household transmissibility, heterogeneity in infectiousness of cases, and the accuracy (given by a set of four probabilities) of case ascertainment.

We also carried out a comprehensive review of household-based studies, which were typically undertaken early in the pandemic. The overwhelming majority of these did not estimate transmission probabilities between individuals, but instead reported crude secondary attack rates (SARs, see Additional file 1: Literature Review for a formal definition) amongst household contacts of initially detected individuals. While this approach is natural in the context of an emerging and rapidly growing pandemic, accurate estimation of transmission intensity allows more general conclusions to be obtained, which can inform public health management strategies. This is because SARs arise as a result of interaction between the biological process of transmission and the socio-demographic structure of a population. Estimation of the transmission probabilities independently of the demographic structure, as presented here, therefore allows more general conclusions to be drawn.

**Figure 1:**
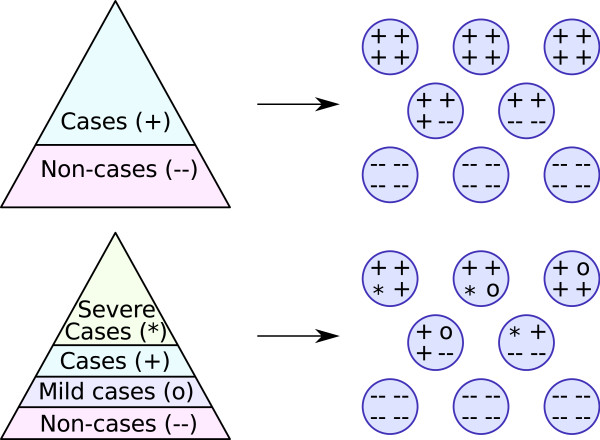
**Non-mathematical explanation of the method used**. Risk pyramids are shown on the left and households on the right. Top: If cases and non-cases are straightforwardly ascertained, then within-household transmission will tend to cluster the cases so that there are either many or few cases within a household. Bottom: The presence of further stratification complicates the picture if information is only available on one outcome, but if full information is available, the clustering of cases by household is still visible and gives an accurate picture of transmission. Case ascertainment is not explicitly represented in this cartoon, but the principle is similar.

The feature that sets our study apart from previous work is the combined use of multiple case definitions. In the majority of situations, public-health investigations of household infections record many observations about the individuals' health and symptoms. Then this rich information is generally converted to a binary outcome (infected or not) according to a strict case definition, with such definitions typically varying between different public-health bodies. Here we develop a methodology that can be applied whenever plausible case definitions form a nested hierarchy as shown in Figure 1, and show how retaining the individual-level heterogeneous data allows us to compute the likely true infections and the errors associated with different case definitions.

## Methods

We now present the protocols used to obtain data during the early phase of the 2009 pandemic. The key features of these protocols will apply in many different outbreak scenarios. These protocols motivated our statistical methodology, which is also described below.

### Data collection

During the initial phase of the pandemic in 2009 in the UK, suspected cases of influenza A(H1N1)pdm09 were reported to the Health Protection Agency (HPA) by general practitioners. Individuals meeting a strict case definition were classified as 'possible' cases of influenza A(H1N1)pdm09 [8]. The case definition included presence of fever or history of fever, and two other specified flu-like symptoms or other severe illness consistent with influenza infection. Epidemiological criteria were included in the case definition in addition to clinical criteria and related to recent travel to high incidence areas and contact with other laboratory confirmed cases of influenza A or influenza A(H1N1)pdm09 infection. Possible cases were prescribed antiviral treatment and had nasal and/or throat swabs taken in order to confirm the diagnosis, which was done using real-time PCR (RT-PCR) methods. All household contacts of laboratory-confirmed cases were subsequently prescribed antiviral prophylaxis, and any symptomatic contacts meeting the case definition were managed as 'possible' cases. Detailed demographic and clinical information regarding suspected cases and the household contacts of laboratory-confirmed cases was collected by the HPA during this early period, known as the containment phase.

In Birmingham, BADGER (Birmingham and District General Practitioner Emergency Rooms - a cooperative of local general practitioners) which currently provides out-of-hours primary care services in the city, were commissioned by the local primary care trusts (health administrative organizations) to set up the Birmingham Flu Service. The staff was initially commissioned to undertake testing for H1N1 of patients referred by the HPA with likely symptoms. Subsequently they undertook the case management of possible cases of H1N1 and their household contacts (in this case using the standard definition of households as individuals sharing living arrangements) including taking nose/throat swab samples and providing antiviral prophylaxis and treatment. This testing involved all suspected cases of H1N1 (and their household contacts) notified to the HPA by all general practitioners (family doctors) within the Birmingham Primary Care Trust area, and as such should sample representatively the H1N1 cases in general population of Birmingham, although there is the possibility of differential reporting by population subgroups. The first laboratory-confirmed case seen by the clinic, with a documented illness onset date, reported becoming unwell on 5 May 2009. The West Midlands region, which covers a broad area of central England consisting of both rural and urban areas (including Birmingham) had its first reported case on 30 April 2009.

On 19 June 2009 several postcode areas in Birmingham were identified as 'hotspots' or areas of sustained community transmission, and on 23 June 2009 Birmingham as a whole was declared a hotspot. Alternative management strategies were adopted from 19 June 2009 onwards: individuals were treated with antivirals on the basis of clinical suspicion rather than laboratory confirmation if they were contacts of a confirmed case. From 26 June 2009 onwards swabbing and contact tracing ceased in hotspot areas and individuals were treated on the basis of clinical suspicion alone. The data presented here are based on information collected by the BADGER Flu Clinic and the HPA regarding the initial laboratory-confirmed cases, and their household contacts, seen by clinic staff. Data include index cases with reported dates of illness onset and antiviral treatment commencement (where reported) between 5 May 2009 and 18 June 2009, before the change in management strategy was announced. Index cases without illness onset dates are also included in the analysis, 45 of whom may or may not have had dates of onset within the above-defined timeframe. There will also have been a number of contacts who would have been identified after 18 June 2009. These cases and contacts may have, therefore, been treated on the basis of clinical suspicion, rather than swabbed. Information regarding 424 initial cases (in 424 separate households) and their 1,612 household contacts was used to generate the data for analysis. Specific information extracted for the purposes of this study included broad postcode area of the households, details regarding presence of symptoms in contacts, whether swabs had been taken (that is, the individual met the case definition) and associated laboratory results.

### Statistical analysis

Our data involve counts of the number of cases in households of different sizes. Appropriate probabilistic models for such 'final-size' data were presented in a paper by Ball [9]. To deal with these mathematically sophisticated models, we make use of Bayesian Markov Chain-Monte Carlo for statistical analysis [10], which was proposed as a method for dealing with household final-size epidemic data by O'Neill and Roberts [11]. To calculate the likelihood requires two pieces of information: the actual number of cases in each household, and a distribution describing the population-level heterogeneity in infectiousness. For the latter of these, we use a Gamma distribution as a simple, parametric choice. For the former, we have no 'gold standard' test that gives the actual number of cases; instead, for each household we have:

*n *Household size.

*k*_3 _**Symptomatic **individuals with at least one symptom suggestive of an acute respiratory infection (ARI).

*k*_2 _**Swabbed **individuals, who should have met the case definition of fever and two or more other symptoms according to the HPA algorithm.

*k*_1 _**Laboratory-confirmed **cases, where PCR testing of the swab returned a positive result.

**Figure 2:**
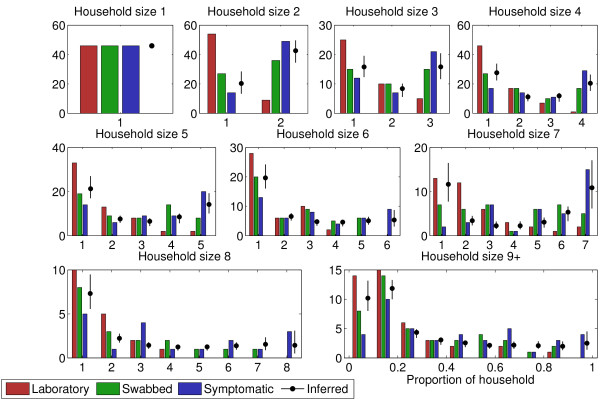
**Final size data**. The final size data (that is, frequency distribution of total number of cases by household size) for different classifications of cases: Laboratory confirmation through PCR; being swabbed (most likely due to meeting HPA ILI diagnostic criteria); having any ARI symptoms; and inferred in the statistical model. Histograms are stratified by household size. ARI, acute respiratory infection; HPA, Health Protection Agency; ILI, Influenza-like illness.

Note that for any individual household, *k*_1 _≤ *k*_2 _≤ *k*_3 _≤ *n*. The histograms showing these data are plotted in Figure 2. The intuition behind our approach is given in Figure 1: within-household transmission gives a distinctive, clustered, pattern to the distribution of cases in households that can be broken by stratification of cases. Use of full information does, however, allow accurate epidemiological information to be obtained. Our full methodology is quite technical and is detailed in Additional file 2: Technical Appendix. The quantities that we estimate (in statistical language, our model parameters) are, however, straightforward to interpret:

*T_n _*Probability of transmission between an infectious and a susceptible individual in a household of size *n *(defined unambiguously in Additional file 2).

*θ *Variance in infectiousness of H1N1pdm09 cases.

*p *Probability that a swab of an H1N1pdm09 case does not return positive.

*q *Probability that a symptomatic H1N1pdm09 case is not swabbed.

*r *Probability that a symptomatic non-H1N1pdm09 individual is swabbed.

*s *Probability that a non-H1N1pdm09 individual has symptoms - this is essentially the baseline prevalence of symptoms indicative of non-H1N1pdm09 ARI.

Perfect case ascertainment therefore corresponds to the situation where *p *and *q *are both zero, and it is obviously also desirable for *r *and *s *to be zero. We check that our methodology arrives at accurate parameter values for several simulated datasets with different parameter values in Additional file 2.

For the recent pandemic, we have made the assumptions that case ascertainment through swabbing and laboratory confirmation does not lead to false positives, and that to acquire immunity or transmit infection individuals must be symptomatic. In terms of the latter assumption, while there is evidence for asymptomatic seroconversion [12-14], our definition of symptomatic cases is particularly inclusive, and could include, for example, individuals with only a sore throat and no fever. Whether seroconversion is possible without even extremely mild symptoms, and if it is, the implications of this for susceptibility and transmissibility, remain unclear. Significant completely asymptomatic acquisition of full immunity would, however, require a different model from that adopted here. We assume that for the nine cases that had a positive swab result but no record of symptoms, there actually were symptoms that were not recorded - asymptomatic individuals were not supposed to be swabbed, and since this is a small proportion of the sample any assumption made does not substantially influence the final results. A further potential source of bias would be other co-circulating respiratory pathogens; however, the expected level of these in Birmingham during late Spring and early Summer is very low, and so for our study period this is unlikely to have been important [15,16].

## Results and discussion

There are three main sets of results from our statistical analysis: estimates of transmission probabilities stratified by household size; an estimate of the population-level heterogeneity of infectiousness; and estimates of probabilities describing case ascertainment.

**Figure 3:**
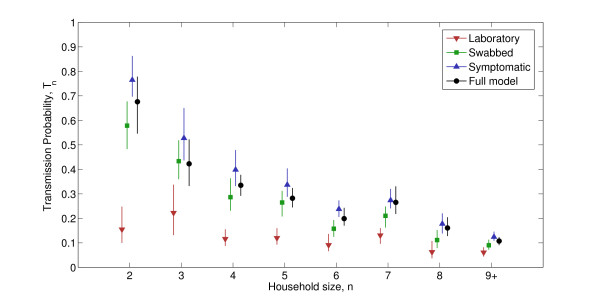
**Transmission probabilities**. Transmission probabilities for different household sizes by three different case definitions and inferred in statistical model, with point estimates and 95% CI shown.

Figure 3 shows the inferred transmission probabilities for the full model, which includes the effects of imperfect case ascertainment (black circles). Also shown are the results that would have been obtained using different case definitions: symptomatic individuals (blue upwards-facing triangles) swabbed individuals (green squares) and individuals with a positive swab result (red downwards-facing triangles). Each of these three definitions has been used previously by other researchers; while some studies presented results using different case definitions, our work is unique in combining different definitions with a model of case ascertainment to provide an explicit estimate of transmission probability. Using the full model, we arrive at a 'true' SAR of 39.7[34.9,44.0]%, compared to 16.0[13.4,18.7]% for PCR, 35.2[31.4,39.1]% for influenza-like illness (ILI), and 51.9[47.5,56.4]% for ARI.

**Figure 4:**
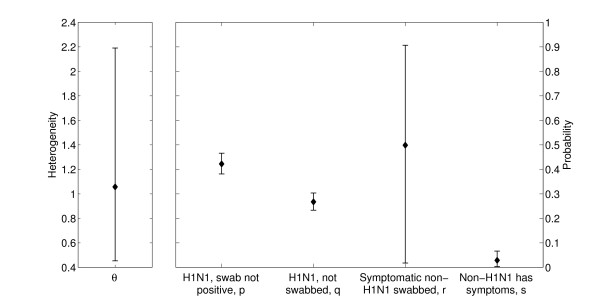
**Other epidemiological parameters**. Left: Heterogeneity, defined as population-level variance in infectiousness. Right: case ascertainment probabilities. Point estimates and 95% CI for the full model are shown for all parameters.

The variance in infectiousness of cases is shown in the left-hand panel of Figure 4. The interpretation of this heterogeneity parameter, *θ*, is in general quite technical; but if it takes the value zero then each case has exactly the same infectiousness, while variability increases with *θ *so that if its value is close to one then the top 10% of cases are more than twenty times as infectious over the total course of their infection as the bottom 10%. While there is a lot of uncertainty in our estimate of *θ*, all of the values in the 95% CI represent significant population-level heterogeneity in infectiousness.

The probabilities relevant to case ascertainment are shown in the right-hand panel of Figure 4. We consider these in turn. Our estimates are that for cases of influenza A(H1N1)pdm09, 27[24,30]% were not swabbed, and of those that were 42[38,47]% did not return a positive swab. This is qualitatively consistent with the serological work of Miller *et al*. [17], although differences in study population means that a quantitative comparison cannot be made. Our estimate for the baseline attack rate with non-H1N1pdm09 ARI over the relevant time period is 3[0.3,7]%, which is again broadly consistent with other work [15,16]. Concerning non-H1N1pdm09 cases of ARI, our estimate of the proportion swabbed contains too much uncertainty to inform policy on the basis of the dataset considered.

Finally, in addition to the original data, Figure 2 shows the inferred distribution of H1N1pdm09 final size-probabilities (black circles). An important feature of these distributions is that they are often bimodal rather than unimodal, that is they look more like the letter 'u' than the letter 'n'. It is this unusual shape that allows for more information to be extracted from household final-size data than is available for crude population-level estimates of prevalence and incidence without household stratification.

## Conclusions

In this study, we have analyzed the household-stratified early infection patterns for pandemic influenza in inner-city Birmingham, UK. We have used modern computationally-intensive statistical methods to fit a realistic model for transmission, and our comprehensive literature search [see Additional file 1] indicated that our approach to modelling the case ascertainment of influenza A(H1N1)pdm09 is novel and provides valuable additional information.

Three key conclusions can be drawn from our work. First, the level of within-household transmission can be estimated directly, despite difficulties in case ascertainment. An estimate of this quantity is important if antivirals are distributed prophylactically to household contacts of cases; if there is little transmission within the household then such a policy is less likely to be effective and vice versa.

Considering our results, we arrive at a 'rule of thumb' for the H1N1pdm09 pandemic that transmissibility lies somewhere between what would be predicted from the HPA definition of ILI (swabbed) and a less specific reporting of ARI symptoms (symptomatic). Our results therefore provide evidence that relying solely on laboratory-confirmed cases is excessively stringent and consistently leads to under-estimation of transmission, as would be expected from serological work [17,18]. An additional consequence of relying on laboratory confirmation is that given this case definition, the transmission probabilities do not decline swiftly as household size increases, while our full model shows a reduction in transmission probability as household size increases, as expected, with the exception of household size seven (we did not find any direct cause for this anomaly). The question of the relative importance of large households for epidemic spread remains significant, and while pre-pandemic analysis of seasonal influenza suggested decline with size [19,20], this was not a consistent observation during the pandemic as seen in [21] and our literature review [see Additional file 1]. Study design may be an important part of variability [21], and our results show that case ascertainment is also relevant.

As part of estimating the transmission process, we also calculated the probability of a false negative PCR result. Forty-two percent of infected cases are estimated to have had a negative laboratory test, which has significant public health importance, and may have been caused by a combination of a number of factors including: problems encountered with taking the swabs [22,23]; swabbing individuals who were not in the early stages of their illness [24]; and potentially swabbing individuals with milder forms of illness. Exploring these factors could also be the focus of future work.

Secondly, our analysis provides additional support for the picture of the recent influenza pandemic as one with highly variable clinical outcomes, including significant numbers of cases who did not meet the HPA's diagnostic criteria, but are likely to have been true cases, and a high variance in the infectiousness of cases, that is there were many cases who were not particularly infectious, while a relatively small minority had an extremely high probability of passing influenza on to their household contacts.

Finally, and most significantly, our approach could be used in future outbreaks as a rapid complement to serological work. Serology provides an important independent test of clinical surveillance methods, but is costly and the correct epidemiological interpretation of an individual's titre is not always clear. Our methods are inexpensive and model the epidemiology of disease transmission directly, giving the potential for an early snapshot of the proportion of cases ascertained.

While we have given certain questions priority in our analysis, as is unavoidable, there are factors that were not captured in our model. We believe that the stratification of cases by age is the most significant omission from our analysis, while other potentially important factors are estimation of between-household transmission and the efficacy of interventions such as encouragement of personal hygiene measures and use of antiviral drugs. In general, inclusion of these additional complexities will lead to stratification of our transmission estimates by age, time to treatment and prophylaxis and so on, in addition to household size, but these may still on average be similar to our unstratified estimates. The expectation from our literature review would be for lower transmission among those given antivirals early and adults than those given antivirals late and children, but the often subtle effects of transmission dynamics mean that this can only be conjectured in the absence of a full analysis.

Ultimately, our ability to extend the model relies on sufficient data being available. Our data are of good quality, but still only contain a finite amount of information. Furthermore, as highlighted there is some missing data on which individuals were managed after 18 June, and therefore treated on the basis of clinical suspicion rather than swabbed. In our review of the literature on household transmission of pandemic influenza [see Additional file 1] we found many studies, involving between them several thousand cases and household contacts, that produced relevant data. Of these, only a small fraction fitted a transmission model to extract generalizable epidemiological conclusions.

The current UK Influenza Preparedness Strategy stresses the need for rapid research early in a pandemic to improve understanding and inform response, and to develop appropriate protocols for such research [1]. We suggest that protocols for collection, sharing and meta-analysis of household data should form part of this preparedness. The data for the studies we found were mostly collected before the end of June 2009. Much of the information collected during these studies, in particular syndromic information and household stratification, was not reported and used at the time. An internationally co-ordinated meta-analysis of household data during July 2009, fitting transmission parameters and adjusting for case definitions so that meaningful comparisons could be made across different demographic and healthcare contexts, could have provided useful information about the pandemic at relatively low cost. In particular, these estimates of disease transmission could be used in a timely fashion to guide changes in public health management strategies, which in England in 2009 were made only in areas where there was evidence of sustained community transmission.

## Abbreviations

ARI: Acute respiratory infection; BADGER: Birmingham and District General Practitioner Emergency Room; HPA: Health Protection Agency; ILI: Influenza-like illness; RT-PCR: Real-time polymerase chain reaction; SAR: Secondary attack rate.

## Competing interests

The authors declare that they have no competing interests.

## Authors' contributions

TH and NI conducted the literature review. NI, FW, OE, GS and BO collected the data. SS designed and developed the database and oversaw the data collection and entry process. TH, JVR and MJK formulated the mathematical model. TH implemented and ran the inference procedure. All authors contributed to the design and writing of the paper. All authors read and approved the final manuscript.

## Pre-publication history

The pre-publication history for this paper can be accessed here:

http://www.biomedcentral.com/1741-7015/10/117/prepub

## Supplementary Material

Additional file 1 **Literature Review**. PDF containing the literature review. Click here for file

Additional file 2 **Technical Appendix**. PDF containing the technical background to the work. Click here for file
