# Supplementary material for: Estimation of outbreak severity and transmissibility: Influenza A(H1N1)pdm09 in households
Source: BMC Med. 2012 Oct 9;10:117. doi: 10.1186/1741-7015-10-117 (PMC3520767; doi:10.1186/1741-7015-10-117)
Supplement: Additional file 1 — Literature Review. PDF containing the literature review. [file 1741-7015-10-117-S1.PDF]

Estimation of outbreak severity and transmissibility: Influenza A(H1N1)pdm09 in households

Literature Review

Thomas House   Nadia Inglis   Joshua V Ross   Fay Wilson
Shakeel Suleman   Obaghe Edeghere   Gillian Smith   Babatunde Olowokure
Matt J Keeling

Methodology

Search

Searches were carried out in Medline, Pubmed and Web of Knowledge using the following basic search strategy: "(influenza OR flu) AND (house OR household OR households OR community OR communities) AND (attack OR transmission)".

Selection

Studies were included that: (1) considered transmission within households, as opposed to other contexts like schools; (2) related to influenza A(H1N1)pdm09, rather than other respiratory pathogens; and (3) either fitted household transmission models, or reported raw household secondary attack rates. Titles and abstracts were screened for relevant articles published (including online-first manuscripts) since the start of 2009 and before February 2011.

Validity assessment

Studies reporting only overall community attack rates, and those from which secondary attack rates could not be calculated were excluded from subsequent review. Studies which did not provide sufficient information in English for the purposes of the review were excluded, as were studies based on epidemics of other influenza strains, in particular seasonal influenza during the same timeframe.

Data abstraction

Two authors (TH and NI) duplicated the abstraction of data into Tables 1 and 2 below. This was done manually by reading the full published text of each article.

Study characteristics

7 studies were identified which fitted household transmission models (shown in Table 1) and 35 which reported raw household secondary attack rates (shown in Table 2).

Interpretation

In this paper, we make a distinction between the Secondary Attack Rate (SAR) and transmission probability, that does not always exist in other papers, but appears to be necessary to remove ambiguity. In Tables 1 and 2, we use the definitions

$$\text{SAR} = \frac{\text{Number of infected household contacts}}{\text{Number of household contacts}} \times 100\% \ ,$$

$$T_n^{a \to b} = \text{Probability of an infectious individual of age } a \text{ transmitting to a susceptible individual of age } b \text{ in a household of size } n\text{, ignoring other household members.}$$

The mathematically unambiguous formula for T is given in Additional File 2.

Overall, the studies found present highly variable numerical estimates for SARs in descriptive studies, and transmission probabilities in model-based studies, making quantitative meta-analysis problematic [1]. This variability is likely to arise from a combination of factors: differences in household size distribution, case ascertainment (i.e. different case definitions used), household composition by age, and the effects of public health interventions. In our analysis, we consider the first two effects, but not the latter two. Therefore, we help to explain a proportion of the heterogeneity in reported household transmissibility of pandemic influenza, but it would require further work (and ideally access to all datasets found during the review) to disentangle the impact of all relevant factors.

Our search also produced several results that did not meet the criteria for inclusion into Tables 1 and 2, but which are clearly relevant for the current study. Girard et al. [2] wrote a broad review of the literature on pandemic influenza touching on epidemiology, virology and vaccination. Boëlle et al. [3] reviewed studies of non-household transmission parameters for pandemic influenza. Fraser et al. [4] use household data to infer probabilities related to asymptomatic transmission and acquisition of 1918 pandemic influenza, which is conceptually a different problem from ours but makes similar use of the structure of household final-size probabilities. Klick et al. [5] consider the design of household studies of infectious disease. Several studies we included considered the impact of pharmaceutical interventions; however, for non-pharmaceutical interventions like face masks and hand washing most studies considered non-pandemic influenza [6, 7, 8] while one reported results for influenza A(H1N1)pdm09 [9].

Table 1: Studies fitting a transmission model to data

**Study***	**Dates (2009)**	**Population**	**Index cases**	**HH contacts**	**Index case def.****	2° **case def.****	**Model fitted, estimates and 95% CIs†**
**1.** Yang et al. [10]	25 Mar – 29 Apr	First US households reported by CDC MMWR.	5	13	PCR	PCR	T ranges between 0.2[0.1,0.4] and 0.3[0.1,0.5] depending on assumptions about missing data.
**2.** Cauchemez et al. [11]	19 Apr – 25 May	US households reported to CDC by state health departments.	216	600	PCR	ARI or ILI	$T_2 = 0.22[0.1, 0.43]$, falling to $T_6 = 0.01[0, 0.06]$ for ARI; $T_2 = 0.14[0.05, 0.29]$, falling to $T_6 = 0.0045[0.0001, 0.03]$ for ILI.
**3.** Sugimoto et al. [12]	25 Apr – 9 Jun	Household contacts of index cases of ILI in youth camp members in Washington State, US.	42	136	ILI+ or ARI	ILI+ or ARI	$T^{y\to y} = 0.108[0.047, 0.231]$, $T^{y\to o} = 0.025[0.008, 0.072]$ for strictest ILI; $T^{y\to y} = 0.406[0.101, 0.805]$, $T^{y\to o} = 0.223[0.049, 0.615]$ for ARI.
**4.** Ghani et al. [13]	27 Apr – 10 Jun	UK 'First Few Hundred' (FF100) dataset collated by the HPA.	193	556	PCR	PCR or ILI	T fitted but numerical value not reported. Raw SAR given as 11.2% for ILI and 8.1% for PCR.
**5.** Cauchemez et al. [14]	27 Apr – 30 May	Elementary school and semirural community in PA, USA.	295	899	ARI	ARI	All index cases are at elementary school. $T_2^{y\to y} \approx 0.32[0.12, 0.65]$, $T_2^{y\to o} = 0.096[0.032, 0.24]$, $T_6^{y\to y} \approx 0.1[0.05, 0.15]$, $T_6^{y\to o} = 0.026[0.015, 0.042]$.
**6.** van Boven et al. [15]	29 Apr – 15 Aug	Cases reported to RIVM, Netherlands, through municipal health authorities.	47	109	PCR	PCR	Model is split into old and young (12 and under). $T^{y\to y} = 0.13[0.0086, 0.42]$, $T^{y\to o} = 0[0, 0.059]$, $T^{o\to y} = 0[0, 0.059]$, $T^{o\to o} = 0.057[0.014, 0.14]$.

**7.** Klick et al. [16]	Apr – Oct	Prospective, randomized, placebo-controlled, double-blind pilot study of households in Hong Kong.	425 individuals in 117 households.		Sero	Sero	For any age a, $T^{a\to y} = 0.15[0.05, 0.28]$, $T^{a\to o} = 0.07[0.00, 0.15]$. Distinction between initial and secondary cases not made explicitly in study design.
**8.** This study.	5 May – 18 Jun	Laboratory-confirmed cases and their household contacts in Birmingham, England.	424	1612	PCR	PCR, ILI or ARI	$T_2 = 0.677[0.548, 0.788]$, $T_6 = 0.2[0.169, 0.239]$ (see Results section for full set of parameter estimates). 'True' SAR is 39.7[34.9,44.0]%

Notes: *all studies above are retrospective unless otherwise stated; **'PCR' means polymerase chain reaction, 'ILI' means diagnosis on rigorous criteria, 'ILI+' means several sets of criteria used, 'ARI' means diagnosis on wider symptomatic criteria, 'Sero' means seroconversion; †≈ is used for estimates read from figures rather than numerical values.

Table 2: Studies reporting a 'raw' secondary attack rate

Study*	Dates**	Population (Design)	Index cases	HH contacts	Index case def.†	2° case def.†	SAR estimate [95% CI if reported] and notes
**1.** Calatayud et al. [17]	15 Apr – 15 May	Household contacts of index cases from school in London (primary and senior school). (Retrospective)	33	Not given	PCR	PCR	17% overall.
**2.** Carcione et al. [18] / **3.** Carcione et al. [19]	29 May – 7 Aug	Household contacts of laboratory-confirmed cases in Western Australia. (Prospective)	595	1466 / 1589	PCR	ILI	15% / 14.5[12.9,16.4]%, decreasing with age.
**4.** Centers for Disease Control and Prevention [20]	29 Jun – 5 Jul	Household contacts (including student groups staying in same house/floor of hotel) of first four laboratory-confirmed cases identified in Kenya. (Retrospective)	4	54	PCR	PCR	26[7,33]% overall. No access to antivirals.
**5.** Chang et al. [21]	Aug – Nov	Household contacts of patients at the National Taiwan University Hospital. (Prospective)	87	223	Lab	Lab	27% overall, reducing with age.
**6.** Cowling et al. [22]	Jul – Aug	Household contacts of individuals presenting with pandemic or seasonal influenza (confirmed by rapid testing) to outpatient clinics across Hong Kong. Serology performed for some cases. (Prospective)	41	115	PCR	PCR, ILI or ARI	8[3,14]% for PCR, 6[3,11]% for ILI, 26[16,36]% for ARI.
**7.** Serres et al. [23]	27 May – 10 Jul	Household contacts of individuals with laboratory-confirmed H1N1 in Quebec, Canada. (Prospective)	35	138	PCR	PCR or ARI	23% for PCR, 53% for ARI.

**8.** Doyle and Hopkins [24]	14 – 30 Jun	Household contacts of boys (index cases) who had attended an outdoor camp, Florida. (Prospective)	43	87	PCR + ILI	ILI	3.5% overall, 14.3% if boys returned home 1 day after symptoms appeared.
**9.** France et al. [25] / **10.** Jackson et al. [26]	8 Apr – 27 May	Household contacts of children (index cases) associated with an outbreak in a New York High School. (Retrospective) / Subset of this population. (Prospective)	222 / 32	702 / 79	PCR + ILI / PCR + Sero	ILI / Sero	11.3[8.8,13.7]% overall, decreasing with age / 19[10,28]% overall.
**11.** Goldstein et al. [27]	mid-Apr – mid-Jun	Households in Milwaukee, US, with one laboratory-confirmed case known to the City of Milwaukee Health Department. (Retrospective)	135	411	PCR	ILI	14[10,19]% of 95 households where the index case did not receive timely oseltamivir.
**12.** Komiya et al. [28]	16 – 31 May	Household contacts of index cases (first laboratory-confirmed case in household) reported to health authorities in Osaka, Japan. Assess effect of post-exposure prophylaxis (PEP). (Prospective)	124	379	PCR	PCR	3.7% overall, 26.1% without PEP.
**13.** Lee et al. [29]	24 Aug – 9 Nov	Korean households with at least one member laboratory-confirmed at Chung-Ang University Yongsan Hospital, Seoul. Households with antiviral prophylaxis excluded. (Restrospective)	199	297	PCR	PCR	27.9% overall, decreasing with age.
**14.** Leung et al. [30]	12 – 23 Jun	Household contacts of laboratory-confirmed cases of H1N1 associated with a secondary school outbreak in Hong Kong. (Prospective)	65	205	PCR	PCR	5.9[2.7,9.1]% overall, decreasing with age. Higher if no antiviral prophylaxis.

**15.** Liu et al. [31]	31 May 2009 – 31 Mar 2010	Household/close contacts of cases (for whom clinical information was available) reported to health authorities in Hangzhou, China. (Retrospective)	658	612	PCR	ILI	8.66% overall.
**16.** Looker et al. [32]	1 May – 31 Aug	Household contacts of laboratory-confirmed cases reported via a GP sentinel surveillance scheme in Victoria, Australia. (Retrospective)	132	351	PCR	ILI	33% overall.
**17.** Loustalot et al. [33]	15 Apr – 13 May	Households of High School Students, San Antonio, Texas. (Retrospective)	78	562	ILI	ILI	3.7% overall, 9.1% in 0-4 year olds.
**18.** Mohamed et al. [34]	Sep – Oct	Household contacts of confirmed H1N1 cases presenting to King Khalid University Clinics, Saudi Arabia. Prospective)	69	432	PCR	ARI	16.9% overall.
**19.** Morgan et al. [35]	15 Apr – 8 May	Household contacts of index cases (first in household with ARI, ILI or confirmed H1N1 infection) in Health Service Region 8, San Antonio, Texas. (Retrospective)	77	264	PCR	PCR, ILI or ARI	4% for PCR, 9% for ILI and 13% for ARI.
**20.** Nishiura and Oshitani [36] / **21.** Nishiura and Oshitani [37]	May 2009 – Feb 2010	Household members of index cases (with laboratory-confirmed H1N1 or ILI) across Japan. (Retrospective)	1547 / 109	4609 / 142	PCR or ILI	PCR or ILI	11.4[10.5,12.3]% overall, decreasing with age / 44.4% unvaccinated children, 11% vaccinated children.
**22.** Odaira et al. [38]	16 May – 5 Jun	Household contacts of index cases (first case of ARI or laboratory-confirmed H1N1 in house) following the first domestic outbreak in Kobe, Japan. (Prospective)	97	303	PCR or ARI	PCR or ARI	4.8% overall, decreasing with age.

**23.** Pang et al. [39]	16 May – 15 Sept	Household and other close contacts of laboratory-confirmed index cases in Beijing, People's Republic of China (Prospective) (Prospective)	613	1228	PCR	PCR	5.3% overall, decreasing with age.
**24.** Papenburg et al. [40]	27 May – 10 Jul	Household members of index cases (first laboratory-confirmed cases in house), Quebec, Canada. All household members swabbed, and all those over age 7 had serum samples taken. (Prospective)	43	119	PCR	PCR, Sero, ILI, ARI	45[35.6,53.5]% overall 29% for ILI, 51% for ARI.
**25.** Pebody et al. [41]	27 Apr – 21 Jun	Household members of index cases (first laboratory-confirmed cases in house) in UK, earliest reported cases from "First Few Hundred Dataset". (Retrospective)	285	761	PCR	PCR, ILI or ARI	8.1[6.4,10.3]% for PCR, 10.5[8.5,12.9]% for ILI, 16.7[14.1,19.6]% for ARI, all decreasing with age. Higher if no antiviral prophylaxis.
**26.** Pedroni et al. [42]	1 Apr – 27 Jun	Household members of laboratory-confirmed cases presenting to emergency departments in Puerto Montt, Los Lagos, Chile. (part Retrospective, part Prospective)	57	245	PCR	ILI	36% overall.
**27.** Peltola et al. [43]	Oct – Nov	Household members of six laboratory-confirmed cases of H1N1 in children under 1.5 yrs presenting to a respiratory infection cohort study clinic in Finland. (Prospective)	6	15	PCR	PCR	73[48,99]% overall.

**28.** Savage et al. [44]	24 Apr – 18 Jun	Household members of laboratory-confirmed cases presenting to 7 public health units in Ontario, Canada. (Prospective)	97	253	Lab	ILI or ARI	10.3[6.8,14.7]% for ILI, 20.2[15.4,25.6]% for ARI, decreasing with age.
**29.** Sikora et al. [45]	30 Apr – 9 Jun	Household members of laboratory-confirmed cases in urban households in Edmonton, Canada. (Prospective)	87	262	PCR	ILI	30.2[12.6,52.2]% overall.
**30.** Skowronski et al. [46]	27 May – 10 Jul	Household contacts of the first laboratory-confirmed case in households in Quebec City, Canada. Considered association between 2008-9 trivalent vaccine and infection during pandemic (H1N1) 2009. One of several studies carried out. (Prospective)	47	120	PCR	PCR, ILI or ARI	35% for PCR, 25.8% for ILI, and 60% for ARI.
**31.** Suess et al. [47]	Apr – Aug	All German households with a laboratory-confirmed case responding to Robert Koch Institute contact. (Restrospective)	36	83	PCR	PCR	18% overall. 26% of the 47 contacts not receiving antiviral prophylaxis. No difference in SAR by age.
**32.** Tandale et al. [48]	Sept – Oct	Household contacts of laboratory-confirmed index cases in Pune, India. (Retrospective)	74	195	PCR	Sero	25.6[19.5,31.8]% overall.
**33.** Teh et al. [49]	30 Apr – 31 Jul	Household contacts of laboratory-confirmed index cases and those with any ILI identified from microbiology records, Melbourne, Victoria, Australia. (Retrospective)	318 PCR / 818 ILI	1331 / 499	PCR or ILI	ILI	23.5% for ILI, 30.6% for PCR, decreasing with age.

**34.** van Gemert et al. [50]	18 May – 3 Jun	Household contacts of randomly selected confirmed index cases reported to Victorian Department of Health, Australia, within specified time period. (Retrospective)	36	122	Lab	ARI	14.8[8.9, 22.3]% overall.
**35.** Vilella et al. [51]	19-27 Jun	Household contacts of a group of Spanish medical students reporting ARI/having laboratory-confirmed H1N1 following a trip to the Dominican republic. (Prospective)	39 PCR / 62 ARI	98 / 137	PCR or ARI	PCR or ARI	2.9% for 1° and 2° ARI, 1% for 1° and 2° PCR.
**36.** This study.	5 May – 18 Jun	Laboratory-confirmed cases and their household contacts in Birmingham, England. (Retrospective)	424	1612	PCR	PCR, ILI or ARI	16.0[13.4,18.7]% for PCR, 35.2[31.4,39.1]% for ILI, 51.9[47.5,56.4]% for ARI.††

Notes: *multiple publications from one datset share a row; **dates are 2009 unless otherwise stated; †'PCR' means polymerase chain reaction, 'ILI' means diagnosis on rigorous criteria, 'ARI' means diagnosis on wider symptomatic criteria, 'Lab' means laboratory techniques, 'Sero' means seroconversion; ††CI in raw SARs calculated from a household-level bootstrap.

References

1. L. L. H. Lau, H. Nishiura, H. Kelly, D. K. M. Ip, G. M. Leung, and B. J. Cowling. Household transmission of 2009 pandemic influenza A(H1N1): a systematic review and meta-analysis. To appear in Epidemiology., 2012.

2. M. P. Girard, J. S. Tam, O. M. Assossou, and M. P. Kieny. The 2009 A (H1N1) influenza virus pandemic: A review. *Vaccine*, 28(31):4895–4902, Dec 2010.

3. P.-Y. Boëlle, S. Ansart, A. Cori, and A.-J. Valleron. Transmission parameters of the A/H1N1 (2009) influenza virus pandemic: a review. *Influenza and Other Respiratory Viruses*, 5(5): 306–316, Mar 2011.

4. C. Fraser, D. A. T. Cummings, D. Klinkenberg, D. S. Burke, and N. M. Ferguson. Influenza transmission in households during the 1918 pandemic. *American Journal of Epidemiology*, pages 1–10, Jul 2011.

5. B. Klick, G. M. Leung, and B. J. Cowling. Optimal design of studies of influenza transmission in households. I: Case-ascertained studies. *Epidemiology and Infection*, pages 1–9, Mar 2011.

6. B. J. Cowling, K.-H. Chan, V. J. Fang, C. K. Y. Cheng, R. O. P. Fung, W. Wai, J. Sin, W. H. Seto, R. Yung, D. W. S. Chu, B. C. F. Chiu, P. W. Y. Lee, M. C. Chiu, H. C. Lee, T. M. Uyeki, P. M. Houck, J. S. M. Peiris, and G. M. Leung. Facemasks and hand hygiene to prevent influenza transmission in households: a cluster randomized trial. *Ann Intern Med*, 151 (7):437–46, Oct 2009.

7. C. R. MacIntyre, S. Cauchemez, D. E. Dwyer, H. Seale, P. Cheung, G. Browne, M. Fasher, J. Wood, Z. Gao, R. Booy, and N. Ferguson. Face mask use and control of respiratory virus transmission in households. *Emerging Infectious Diseases*, 15(2):233–41, Feb 2009.

8. E. L. Larson, Y. hui Ferng, J. Wong-McLoughlin, S. Wang, M. Haber, and S. S. Morse. Impact of non-pharmaceutical interventions on URIs and influenza in crowded, urban households. *Public Health Rep*, 125(2):178–91, Jan 2010.

9. J. M. Simmerman, P. Suntarattiwong, J. Levy, R. G. Jarman, S. Kaewchana, R. V. Gibbons, B. J. Cowling, W. Sanasuttipun, S. A. Maloney, T. M. Uyeki, L. Kamimoto, and T. Chotipitayasunondh. Findings from a household randomized controlled trial of hand washing and face masks to reduce influenza transmission in Bangkok, Thailand. *Influenza and Other Respiratory Viruses*, 5(4):256–267, Feb 2011.

10. Y. Yang, J. D. Sugimoto, M. E. Halloran, N. E. Basta, D. L. Chao, L. Matrajt, G. Potter, E. Kenah, and I. M. Longini. The transmissibility and control of pandemic influenza A (H1N1) virus. *Science*, 326(5953):729–33, Oct 2009.

11. S. Cauchemez, C. A. Donnelly, C. Reed, A. C. Ghani, C. Fraser, C. K. Kent, L. Finelli, and N. M. Ferguson. Household transmission of 2009 pandemic influenza A (H1N1) virus in the United States. *N Engl J Med*, 361(27):2619–27, Dec 2009.

12. J. D. Sugimoto, N. N. Borse, M. L. Ta, L. J. Stockman, G. E. Fischer, Y. Yang, M. E. Halloran, I. M. Longini, and J. S. Duchin. The effect of age on transmission of 2009 pandemic influenza A (H1N1) in a camp and associated households. *Epidemiology*, 22(2):180–7, Mar 2011.

13. A. Ghani, M. Baguelin, J. Griffin, S. Flasche, A. J. V. Hoek, S. Cauchemez, C. Donnelly, C. Robertson, M. White, J. Truscott, C. Fraser, T. Garske, P. White, S. Leach, I. Hall, H. Jenkins, N. Ferguson, and B. Cooper. The early transmission dynamics of H1N1pdm influenza in the United Kingdom. *PLoS Curr*, 2:RRN1130, Jun 2010.

14. S. Cauchemez, A. Bhattarai, T. L. Marchbanks, R. P. Fagan, S. Ostroff, N. M. Ferguson, D. Swerdlow, S. V. Sodha, M. E. Moll, F. J. Angulo, R. Palekar, W. R. Archer, and L. Finelli. Role of social networks in shaping disease transmission during a community outbreak of 2009 H1N1 pandemic influenza. *Proceedings of the National Academy of Sciences*, 108(7):2825–2830, Feb 2011.

15. M. van Boven, T. Donker, M. V. D. Lubben, R. B. V. Gageldonk-Lafeber, D. E. T. Beest, M. Koopmans, A. Meijer, A. Timen, C. Swaan, A. Dalhuijsen, S. Hahné, A. V. D. Hoek, P. Teunis, M. A. B. V. D. Sande, J. Wallinga, and Y. Yang. Transmission of novel influenza A(H1N1) in households with post-exposure antiviral prophylaxis. *PLoS ONE*, 5(7):e11442, Jul 2010.

16. B. Klick, H. Nishiura, S. Ng, V. J. Fang, G. M. Leung, J. S. Peiris, and B. J. Cowling. Transmissibility of seasonal and pandemic influenza in a cohort of households in Hong Kong in 2009. *Epidemiology*, 22(6):793–6, Nov 2011.

17. L. Calatayud, S. Kurkela, P. E. Neave, A. Brock, S. Perkins, M. Zuckerman, M. Sudhanva, A. Bermingham, J. Ellis, R. Pebody, M. Catchpole, R. Heathcock, and H. Maguire. Pandemic (H1N1) 2009 virus outbreak in a school in London, April-May 2009: an observational study. *Epidemiology and Infection*, 138(2):183–91, Feb 2010.

18. D. Carcione, C. Giele, L. S. Goggin, K. S. Kwan, D. W. Smith, G. K. Dowse, D. B. Mak, and P. Effler. Association between 2009 seasonal influenza vaccine and influenza-like illness during the 2009 pandemic: preliminary results of a large household transmission study in Western Australia. *Euro Surveill*, 15(28), Jan 2010.

19. D. Carcione, C. M. Giele, L. S. Goggin, K. S. Kwan, D. W. Smith, G. K. Dowse, D. B. Mak, and P. Effler. Secondary attack rate of pandemic influenza A(H1N1) 2009 in Western Australian households, 29 May-7 August 2009. *Euro Surveill*, 16(3), Jan 2011.

20. Centers for Disease Control and Prevention. Introduction and transmission of 2009 pandemic influenza A (H1N1) Virus–Kenya, June-July 2009. *MMWR – Morbidity and Mortality Weekly Report*, 58(41):1143–6, Oct 2009.

21. L.-Y. Chang, W.-H. Chen, C.-Y. Lu, P.-L. Shao, T.-Y. Fan, A.-L. Cheng, and L.-M. Huang. Household transmission of pandemic (H1N1) 2009 virus, Taiwan. *Emerging Infectious Diseases*, 17(10):1928–31, Oct 2011.

22. B. J. Cowling, K. H. Chan, V. J. Fang, L. L. H. Lau, H. C. So, R. O. P. Fung, E. S. K. Ma, A. S. K. Kwong, C.-W. Chan, W. W. S. Tsui, H.-Y. Ngai, D. W. S. Chu, P. W. Y. Lee, M.-C. Chiu, G. M. Leung, and J. S. M. Peiris. Comparative epidemiology of pandemic and seasonal influenza A in households. *N Engl J Med*, 362(23):2175–84, Jun 2010.

23. G. D. Serres, I. Rouleau, M. E. Hamelin, C. Quach, D. Skowronski, L. Flamand, N. Boulianne, Y. Li, J. Carbonneau, A. Bourgault, M. Couillard, H. Charest, and G. Boivin. Contagious period for pandemic (H1N1) 2009. *Emerging Infectious Diseases*, 16(5):783–8, May 2010.

24. T. J. Doyle and R. S. Hopkins. Low secondary transmission of 2009 pandemic influenza A (H1N1) in households following an outbreak at a summer camp: relationship to timing of exposure. *Epidemiology and Infection*, 139(01):45–51, Jan 2011.

25. A. M. France, M. Jackson, S. Schrag, M. Lynch, C. Zimmerman, M. Biggerstaff, and J. Hadler. Household transmission of 2009 influenza A (H1N1) virus after a school-based outbreak in New York City, April–May 2009. *The Journal of Infectious Diseases*, 201(7):984–992, Apr 2010.

26. M. L. Jackson, A. M. France, K. Hancock, X. Lu, V. Veguilla, H. Sun, F. Liu, J. Hadler, B. H. Harcourt, D. H. Esposito, C. M. Zimmerman, J. M. Katz, A. M. Fry, and S. J. Schrag. Serologically confirmed household transmission of 2009 pandemic influenza A (H1N1) virus during the first pandemic wave–New York City, April-May 2009. *Clin Infect Dis*, 53(5):455–462, Sep 2011.

27. E. Goldstein, B. J. Cowling, J. J. O'hagan, L. Danon, V. J. Fang, A. Hagy, J. C. Miller, D. Reshef, J. Robins, P. Biedrzycki, and M. Lipsitch. Oseltamivir for treatment and prevention of pandemic influenza A/H1N1 virus infection in households, Milwaukee, 2009. *BMC infectious diseases*, 10(1):211, Jan 2010.

28. N. Komiya, Y. Gu, H. Kamiya, Y. Yahata, Y. Yasui, K. Taniguchi, and N. Okabe. Household transmission of pandemic 2009 influenza A (H1N1) virus in Osaka, Japan in May 2009. *J Infection*, 61(4):284–288, Oct 2010.

29. D. H. Lee, C. W. Kim, J.-H. Kim, J. S. Lee, M. K. Lee, J. C. Choi, B. W. Choi, S.-H. Choi, and J.-W. Chung. Risk factors for laboratory-confirmed household transmission of pandemic H1N1 2009 infection. *American Journal of Infection Control*, 38(10):e43–5, Dec 2010.

30. Y. H. Leung, M. P. Li, and S. K. Chuang. A school outbreak of pandemic (H1N1) 2009 infection: assessment of secondary household transmission and the protective role of Oseltamivir. *Epidemiology and Infection*, 139(01):41–44, Jan 2011.

31. S.-L. Liu, Z.-R. Zhang, C. Wang, Y. Dong, L.-B. Cui, X.-H. Yang, Z. Sun, J. Wang, J. Chen, R.-J. Huang, F. Miao, B. Ruan, L. Xie, H.-X. He, and J. Deng. 2009 pandemic characteristics and controlling experiences of influenza H1N1 virus 1 year after the inception in Hangzhou, China. *J Med Virol*, 82(12):1985–95, Dec 2010.

32. C. Looker, K. Carville, K. Grant, H. Kelly, and J. Brown. Influenza A (H1N1) in Victoria, Australia: A community case series and analysis of household transmission. *PLoS ONE*, 5 (10):e13702, Oct 2010.

33. F. Loustalot, B. J. Silk, A. Gaither, T. Shim, M. Lamias, F. Dawood, O. W. Morgan, D. Fishbein, S. Guerra, J. R. Verani, S. A. Carlson, V. P. Fonseca, and S. J. Olsen. Household transmission of 2009 pandemic influenza A (H1N1) and nonpharmaceutical interventions among households of high school students in San Antonio, Texas. *Clin Infect Dis*, 52 Suppl 1:S146–53, Jan 2011.

34. A. G. Mohamed, A. A. Binsaeed, H. Al-Habib, and H. Al-Saif. Communicability of H1N1 and seasonal influenza among household contacts of cases in large families. *Influenza and Other Respiratory Viruses*, Nov 2011. doi:10.1111/j.1750-2659.2011.00308.x.

35. O. W. Morgan, S. Parks, T. Shim, P. A. Blevins, P. M. Lucas, R. Sanchez, N. Walea, F. Loustalot, M. R. Duffy, M. J. Shim, S. Guerra, F. Guerra, G. Mills, J. Verani, B. Alsip, S. Lindstrom, B. Shu, S. Emery, A. L. Cohen, M. Menon, A. M. Fry, F. Dawood, V. P. Fonseca, and S. J. Olsen. Household transmission of pandemic (H1N1) 2009, San Antonio, Texas, USA, April-May 2009. *Emerging Infectious Diseases*, 16(4):631–7, Apr 2010.

36. H. Nishiura and H. Oshitani. Household transmission of influenza (H1N1-2009) in Japan: Age-specificity and reduction of household transmission risk by Zanamivir treatment. *J Int Med Res*, 39(2):619–28, Jan 2011.

37. H. Nishiura and H. Oshitani. Effects of vaccination against pandemic (H1N1) 2009 among Japanese children. *Emerging Infectious Diseases*, 17(4):746–7, Apr 2011.

38. F. Odaira, H. Takahashi, T. Toyokawa, Y. Tsuchihashi, T. Kodama, Y. Yahata, T. Sunagawa, K. Taniguchi, and N. Okabe. Assessment of secondary attack rate and effectiveness of antiviral prophylaxis among household contacts in an influenza A(H1N1)v outbreak in Kobe, Japan, May-June 2009. *Euro Surveill*, 14(35), Jan 2009.

39. X. Pang, P. Yang, S. Li, L. Zhang, L. Tian, Y. Li, B. Liu, Y. Zhang, B. Liu, R. Huang, X. Li, and Q. Wang. Pandemic (H1N1) 2009 among quarantined close contacts, Beijing, People's Republic of China. *Emerging Infectious Diseases*, 17(10):1824–30, Oct 2011.

40. J. Papenburg, M. Baz, M.-E. Hamelin, C. Rheaume, J. Carbonneau, M. Ouakki, I. Rouleau, I. Hardy, D. Skowronski, M. Roger, H. Charest, G. D. Serres, and G. Boivin. Household transmission of the 2009 pandemic a/h1n1 influenza virus: Elevated laboratory-confirmed secondary attack rates and evidence of asymptomatic infections. *Clinical Infectious Diseases*, 51:1033–1041(9), 1 November 2010. doi: doi:10.1086/656582.

41. R. G. Pebody, R. Harris, G. Kafatos, M. Chamberland, C. Campbell, J. S. Nguyen-Van-Tam, E. McLean, N. Andrews, P. J. White, E. Wynne-Evans, J. Green, J. Ellis, T. Wreghitt, S. Bracebridge, C. Ihekweazu, I. Oliver, G. Smith, C. Hawkins, R. Salmon, B. Smyth, J. McMenamin, M. Zambon, N. Phin, and J. M. Watson. Use of antiviral drugs to reduce household transmission of pandemic (H1N1) 2009, United Kingdom. *Emerging Infectious Diseases*, 17(6):990–9, Jun 2011.

42. E. Pedroni, M. Garcia, V. Espinola, A. Guerrero, C. Gonzalez, A. Olea, M. Calvo, B. Martorell, M. Winkler, M. Carrasco, J. Vergara, J. Ulloa, A. Carrazana, O. Mujica, J. Villarroel, M. Labrana, M. Vargas, P. Gonzalez, L. Caceres, C. Zamorano, R. Momberg, G. Munoz, J. Rocco, V. Bosque, A. Gallardo, J. Elgueta, and J. Vega. Outbreak of 2009 pandemic influenza A(H1N1), Los Lagos, Chile, April-June 2009. *Euro Surveillance*, 15(1), Jan 2010.

43. V. Peltola, T. Teros-Jaakkola, M. Rulli, L. Toivonen, E. Broberg, M. Waris, and J. Mertsola. Pandemic influenza a (H1N1) virus in households with young children. *Influenza and Other Respiratory Viruses*, Sep 2011. doi:10.1111/j.1750-2659.2011.00289.x.

44. R. Savage, M. Whelan, I. Johnson, E. Rea, M. Lafreniere, L. C. Rosella, F. Lam, T. Badiani, A.-L. Winter, D. J. Carr, C. Frenette, M. Horn, K. Dooling, M. Varia, A.-M. Holt, V. Sunil, C. Grift, E. Paget, M. King, J. Barbaro, and N. S. Crowcroft. Assessing secondary attack rates among household contacts at the beginning of the influenza A (H1N1) pandemic in Ontario, Canada, April-June 2009: A prospective, observational study. *BMC public health*, 11(1):234, Jan 2011.

45. C. Sikora, S. Fan, R. Golonka, D. Sturtevant, J. Gratrix, B. E. Lee, J. Jaipaul, and M. Johnson. Transmission of pandemic influenza A (H1N1) 2009 within households: Edmonton, Canada. *Journal of Clinical Virology*, 49(2):90–93, Oct 2010.

46. D. M. Skowronski, G. D. Serres, N. S. Crowcroft, N. Z. Janjua, N. Boulianne, T. S. Hottes, L. C. Rosella, J. A. Dickinson, R. Gilca, P. Sethi, N. Ouhoummane, D. J. Willison, I. Rouleau, M. Petric, K. Fonseca, S. J. Drews, A. Rebbapragada, H. Charest, M.-E. Hamelin, G. Boivin, J. L. Gardy, Y. Li, T. L. Kwindt, D. M. Patrick, R. C. Brunham, and C. S. Team. Association between the 2008-09 seasonal influenza vaccine and pandemic H1N1 illness during Spring-Summer 2009: four observational studies from Canada. *PLoS Med*, 7(4):e1000258, Apr 2010.

47. T. Suess, U. Buchholz, S. Dupke, R. Grunow, M. A. D. Heiden, A. Heider, B. Biere, B. Schweiger, W. Haas, and G. Krause. Shedding and transmission of novel influenza virus A/H1N1 infection in households–Germany, 2009. *American Journal of Epidemiology*, 171(11): 1157–1164, Jun 2010.

48. B. V. Tandale, S. D. Pawar, Y. K. Gurav, M. S. Chadha, S. S. Koratkar, V. N. Shelke, and A. C. Mishra. Seroepidemiology of pandemic influenza A (H1N1) 2009 virus infections in Pune, India. *BMC infectious diseases*, 10:255, Jan 2010.

49. B. Teh, K. Olsen, J. Black, A. C. Cheng, C. Aboltins, K. Bull, P. D. R. Johnson, M. L. Grayson, and J. Torresi. Impact of swine influenza and quarantine measures on patients and households during the H1N1/09 pandemic. *Scandinavian Journal of Infectious Diseases*, Nov 2011. doi:10.3109/00365548.2011.631572.

50. C. van Gemert, M. Hellard, E. S. McBryde, J. Fielding, T. Spelman, N. Higgins, R. Lester, H. Vally, and I. Bergeri. Intrahousehold transmission of pandemic (H1N1) 2009 virus, Victoria, Australia. *Emerging Infectious Diseases*, 17(9):1599–607, Sep 2011.

51. A. Vilella, B. Serrano, M. A. Marcos, A. Serradesanferm, J. Mensa, E. Hayes, A. Anton, J. Rios, T. Pumarola, and A. Trilla. Pandemic influenza A(H1N1) outbreak among a group of medical students who traveled to the Dominican Republic. *Journal of Travel Medicine*, 19(1): 9–14, Dec 2011.
