# Supplementary material for: Estimation of outbreak severity and transmissibility: Influenza A(H1N1)pdm09 in households
Source: BMC Med. 2012 Oct 9;10:117. doi: 10.1186/1741-7015-10-117 (PMC3520767; doi:10.1186/1741-7015-10-117)
Supplement: Additional file 2 — Technical Appendix. PDF containing the technical background to the work. [file 1741-7015-10-117-S2.PDF]

Estimation of outbreak severity and transmissibility: Influenza A(H1N1)pdm09 in households

Technical Appendix

Thomas House Nadia Inglis Joshua V Ross Fay Wilson
Shakeel Suleman Obaghe Edeghere Gillian Smith Babatunde Olowokure
Matt J Keeling

This supplement contains the information needed to reproduce the results in the main paper, but which are mainly of technical rather than medical interest. First, we present equations for our model likelihood, which is built up from several components. Secondly, we define our MCMC sampling algorithm. Thirdly, we describe some of the results more technically than in the main paper.

1 Likelihood

1.1 Household final size

Consider a household of size n, where individuals are either susceptible, infectious or removed. Each susceptible household member experiences a force of infection λ_n per infectious household member, and each infectious individual draws their infectious period from a distribution with Laplace transform ϕ. Then the probability p_k of observing k cases given one initial case is given by a solution to the equation

$$\sum_{k=1}^{l} \frac{\binom{l-1}{k-1} p_k}{\binom{n-1}{k-1}\left(\phi\left(\lambda_n \frac{n-l}{n-1}\right)\right)^k} = 1 \ , \tag{1}$$

which is a special form of the results first derived in [1]. Here and throughout this work, binomial coefficients are given respectively by

$$\binom{n}{m} = \frac{n!}{m!(n-m)!} \ . \tag{2}$$

In equation (1), the Laplace transform of the recovery time distribution must be defined. Our choice for the recovery time distribution is a gamma distribution with unit mean. Note that the mean of the infectious period does not affect the result (1), and that PDF and Laplace transform of this distribution are given by

$$f(t) = \frac{t^{-1+1/\theta} e^{-t/\theta}}{\theta^{1/\theta}\Gamma(1/\theta)} \ , \qquad \phi(s) = (1+\theta s)^{-1/\theta} \ . \tag{3}$$

This means that ϕ is numerically quick to evaluate, while the variance of the distribution θ still allows appropriate model flexibility. Since $\lambda_n \in [0, \infty)$, a parameterisation choice that we make is to work with probabilities of transmission

$$T_n = 1 - (1 + \theta\lambda_n)^{-1/\theta} \in [0, 1] \ . \tag{4}$$

This expression mathematically defines what is meant by a transmission probability in the main text. We write

$$P(k|n, T_n, \theta) = \Pr(k \text{ cases in a household of size } n \text{ given 1 initial case}) \ , \tag{5}$$

which can be calculated from (1), (3) and (4).

1.2 False positives and negatives

We assume four possibilities for false positive and negative results:

p	Probability that a swab of an H1N1pdm09 case does not return positive.
q	Probability that a symptomatic H1N1pdm09 case is not swabbed.
r	Probability that a symptomatic non-H1N1pdm09 individual is swabbed.
s	Probability that a non-H1N1pdm09 individual has symptoms – this is essentially the baseline prevalence of symptoms indicative of non-H1N1pdm09 ARI.

For a particular household i, suppose we observe the following:

n_i	Household Size
k_i^3	Symptomatic individuals ($k_i^3 \leq n_i$)
k_i^2	Swabbed individuals ($k_i^2 \leq k_i^3$)
k_i^1	Swab returned positive ($k_i^1 \leq k_i^2$)
k_i	True cases ($k_i^1 \leq k_i \leq k_i^3$)
l_i	Swabbed individuals without H1N1 ($l_i \leq k_i^2$)

Then the probability of this observation in the model is

$$\begin{aligned} P(k_i^3, k_i^2, k_i^1, k_i, l_i | n_i, T_{n_i}, \theta, p, q, r, s) = {} & P(k|n_i, T_{n_i}, \theta) \times \\ & B\left(k_i^3 - k_i | n_i - k_i, s\right) \times \\ & B\left(l_i | k_i^3 - k_i, r\right) \times \\ & B\left(k_i^2 - l_i | k_i, 1 - q\right) \times \\ & B\left(k_i^1 | k_i^2 - l_i, 1 - p\right) \ , \end{aligned} \tag{6}$$

where B is the binomial PDF

$$B(m|n, \pi) = \binom{n}{m} \pi^m (1 - \pi)^{n-m} \ . \tag{7}$$

In practice, the data needed to calculate this probability are not available, meaning slightly more work is needed.

1.3 Full likelihood

In the expression (6) above, k_i and l_i are not known. Additionally, households are only present in the study if $k_i^1 \geq 1$. For a set of N households indexed by i where we do know k_i^3, k_i^2 and k_i^1, we can write down an appropriate likelihood of data given model parameters given data:

$$\mathcal{L}\left(\{k_i^1, k_i^2, k_i^1\}|\{n_i\}, \{T_n\}, \theta, p, q, r, s\right) = \prod_{i=1}^{N} \frac{\sum_{k_i=k_i^1}^{k_i^3} \sum_{l_i=\max(0,k_i^2-k_i)}^{\min(k_i^3-k_i,k_i^2-k_i^1)} P(k_i^3, k_i^2, k_i^1, k_i, l_i|n_i, T_{n_i}, \theta, p, q, r, s)}{1 - Q_{n_i}} \ , \quad (8)$$

for $P(k_i^3, k_i^2, k_i^1, k_i, l_i|n_i, T_{n_i}, \theta, p, q, r, s)$ given in (6), and probability of returning no positive swabs given by

$$\begin{aligned} Q_n &= \sum_{k=1}^{n} \sum_{k''=k}^{n} \sum_{l=0}^{k''-k} \sum_{k'=l}^{k+l} P(k'', k', 0, k, l|n) \\ &= \sum_{k=1}^{n} P(k|n, T_n, \theta) \sum_{j=0}^{k} B(j|k, 1-q) B(j|j, p) \ , \end{aligned} \quad (9)$$

which ensures that the probabilities of all possible observations sum to one.

2 MCMC Methodology

The method of inference is Bayesian MCMC [2, 3]. Sampling is via Metropolis-Hastings with sequential proposal from a Gaussian distribution of variance shown in Table S1. We take a burn-in of 10^4 samples (where a sample is a full set of parameter proposals) and then take the next 10^6 samples, thinned by a factor of 10^2 to give 10^4 independent samples from the posterior. Priors are shown in Table S1.

3 Results

The full trace plots and posteriors are shown for real data are shown in Figures S1 and S2 below. We also carried out a simulation study, with the results shown in Figures S3 and S4. The trace plots show good mixing, and hence independence of the posterior samples, while the simulation study shows that point estimates and credible intervals can be extracted accurately from appropriate data.

The numerical values inferred for real data are shown in Tables S1 and S2. The interpretation of these is largely discussed in the main paper. The technical interpretation of main Figure 1, and Figure S5 is worth considering. Shown as black dots are the point estimates and credible intervals for the values of p_k as defined above in (1). The simulated data shown as grey bars are finite, and often quite small, samples from the true probability mass function, so it does not reflect badly on the inference procedure if the CI in p_k does not overlap with the top of these bars.

References

[1] F. Ball. A unified approach to the distribution of total size and total area under the trajectory of infectives in epidemic models. *Advances in Applied Probability*, 18(2):289–310, 1986.

[2] W. R. Gilks, S. Richardson, and D. J. Spiegelhalter. *Markov Chain Monte Carlo in Practice*. Chapman and Hall/CRC, 1995.

[3] P. O'Neill and G. Roberts. Bayesian inference for partially observed stochastic epidemics. *J Roy Stat Soc A*, 162:121–129, Jan 1999.

Parameter	Prior	Sampling variance	Posterior Median	Posterior 95% Credible Interval
T_2	$U(0,1)$	0.01	0.677	$[0.548, 0.788]$
T_3	$U(0,1)$	0.01	0.423	$[0.346, 0.508]$
T_4	$U(0,1)$	0.01	0.331	$[0.285, 0.382]$
T_5	$U(0,1)$	0.01	0.286	$[0.247, 0.331]$
T_6	$U(0,1)$	0.01	0.2	$[0.169, 0.239]$
T_7	$U(0,1)$	0.01	0.262	$[0.213, 0.322]$
T_8	$U(0,1)$	0.01	0.162	$[0.125, 0.205]$
T_9	$U(0,1)$	0.01	0.107	$[0.0915, 0.123]$
θ	$U(0,10)$	0.1	1.3	$[0.48, 2.75]$
p	$U(0,1)$	0.01	0.424	$[0.381, 0.468]$
q	$U(0,1)$	0.01	0.268	$[0.236, 0.301]$
r	$U(0,1)$	0.05	0.461	$[0.0521, 0.913]$
s	$U(0,1)$	0.01	0.0299	$[0.00292, 0.0714]$

Table S1: Details of the parameters inferred in the full model from real data.

Parameter	Laboratory Posterior		Diagnosed Posterior		Symptomatic Posterior	
	Median	95% CI	Median	95% CI	Median	95% CI
T_2	0.156	$[0.0858, 0.249]$	0.564	$[0.454, 0.673]$	0.771	$[0.669, 0.855]$
T_3	0.216	$[0.141, 0.316]$	0.427	$[0.335, 0.534]$	0.518	$[0.415, 0.63]$
T_4	0.119	$[0.0853, 0.161]$	0.281	$[0.227, 0.344]$	0.399	$[0.342, 0.468]$
T_5	0.122	$[0.091, 0.163]$	0.26	$[0.213, 0.313]$	0.338	$[0.282, 0.404]$
T_6	0.0943	$[0.0674, 0.128]$	0.157	$[0.125, 0.196]$	0.239	$[0.203, 0.282]$
T_7	0.13	$[0.1, 0.169]$	0.205	$[0.169, 0.248]$	0.275	$[0.236, 0.32]$
T_8	0.0594	$[0.0329, 0.0985]$	0.11	$[0.0764, 0.155]$	0.18	$[0.139, 0.231]$
T_9	0.0592	$[0.0435, 0.0798]$	0.0892	$[0.0714, 0.112]$	0.123	$[0.104, 0.147]$
θ	0.413	$[0.0204, 1.14]$	0.454	$[0.0931, 0.921]$	0.4	$[0.111, 0.763]$

Table S2: Details of the parameters inferred in the restricted models from real data. Priors and sampling variances are as above.

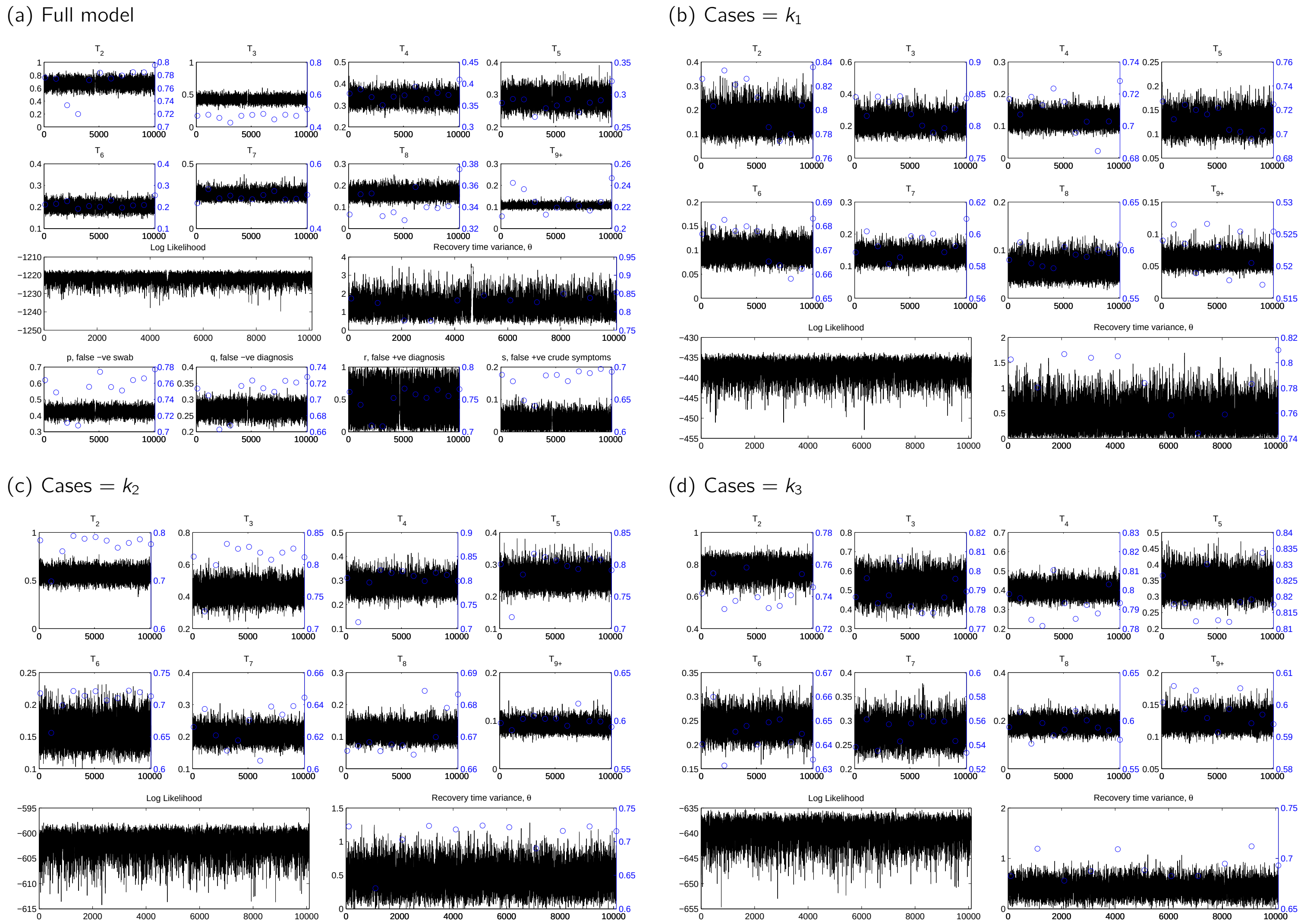

Figure S1: Post-thinning trace plots for real data. Traces are shown as black lines, with values from the left axis and mean acceptance rates as blue circles with values from the right axis.

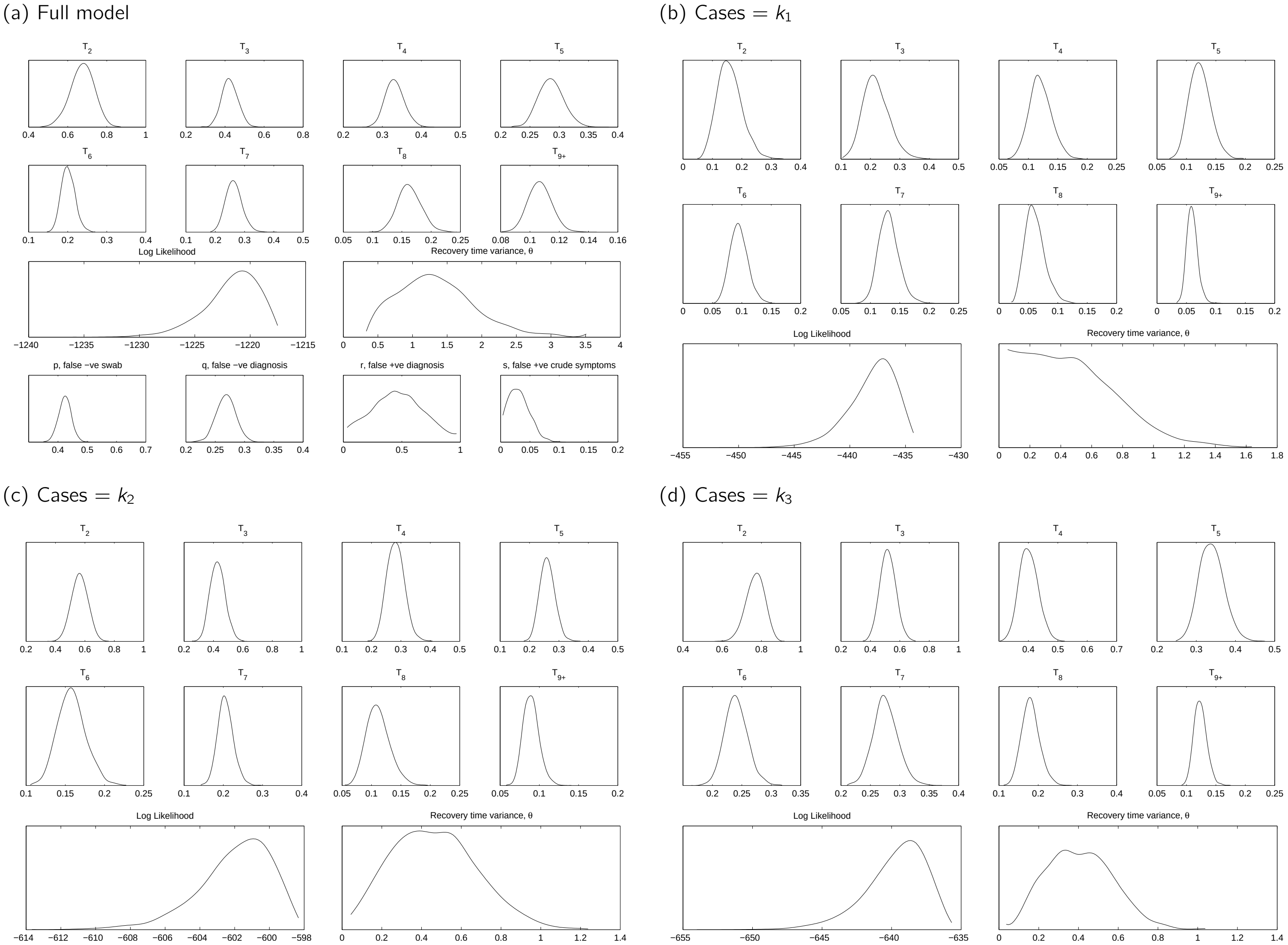

Figure S2: Smoothed posteriors for real data.

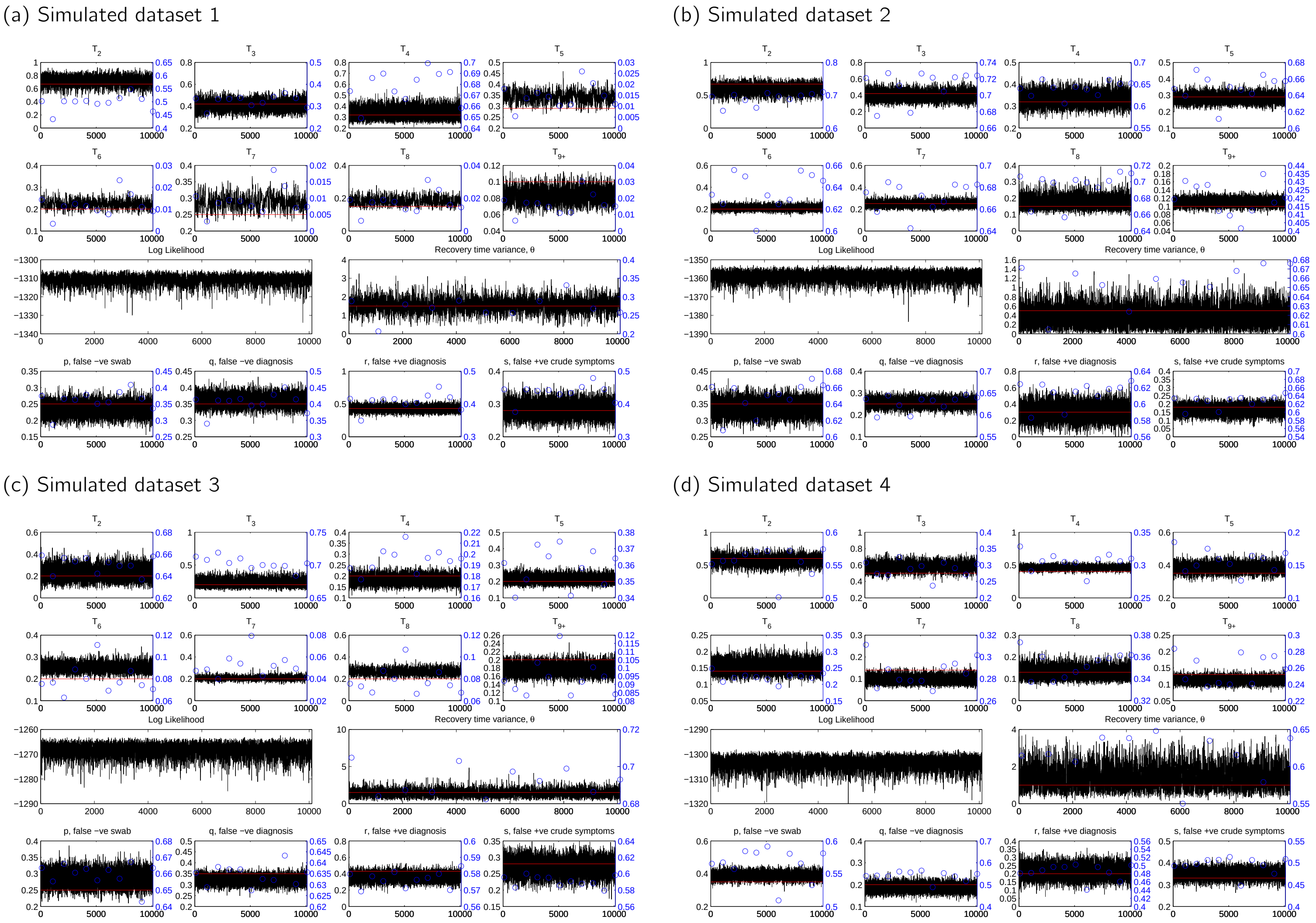

Figure S3: Post-thinning trace plots for simulated data. Traces are shown as black lines, with values from the left axis and mean acceptance rates as blue circles with values from the right axis. Values used in simulation are shown as red lines with values from the left axis.

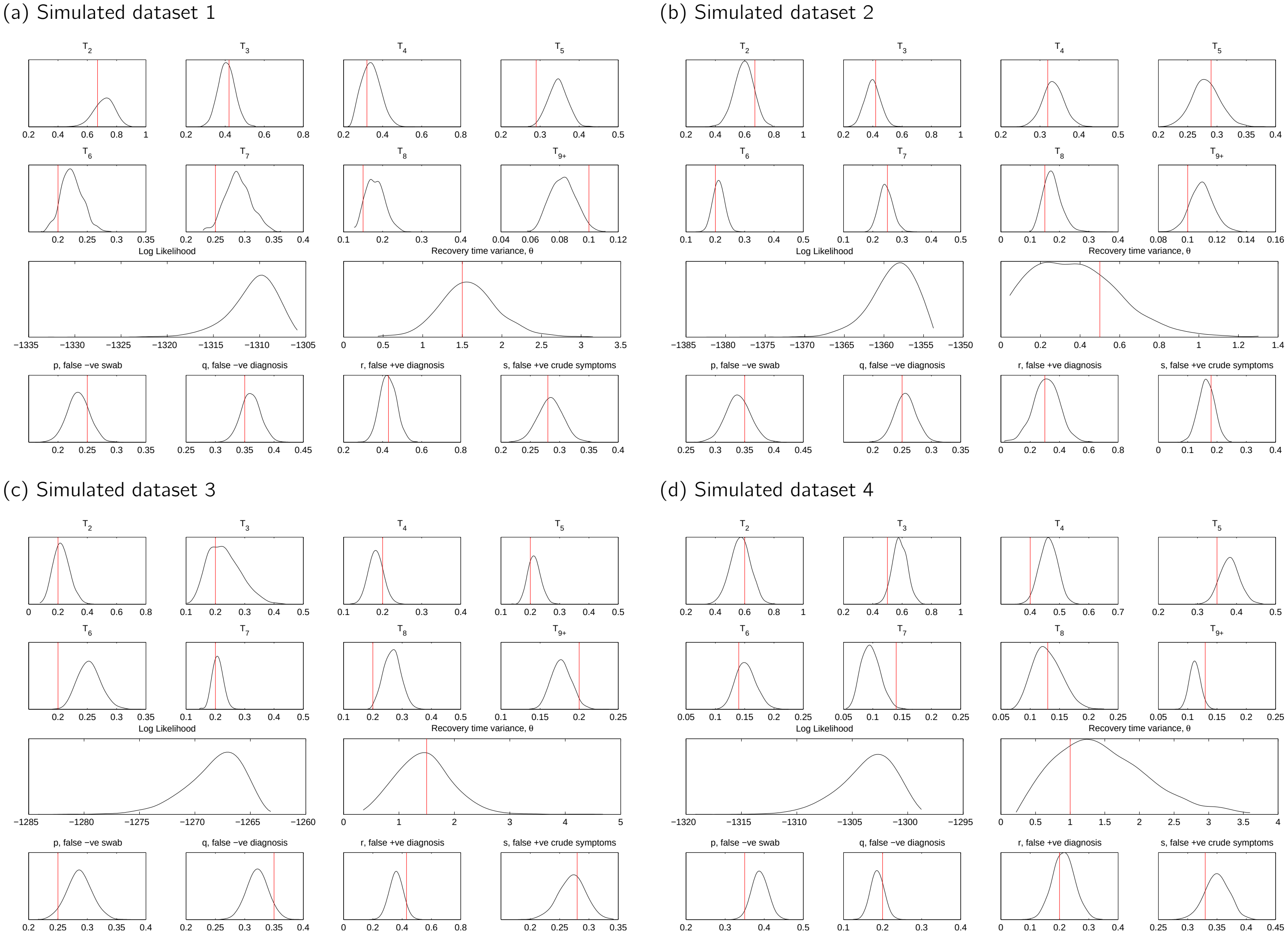

Figure S4: Smoothed posteriors for simulated data. Values used in simulation are shown as red lines.

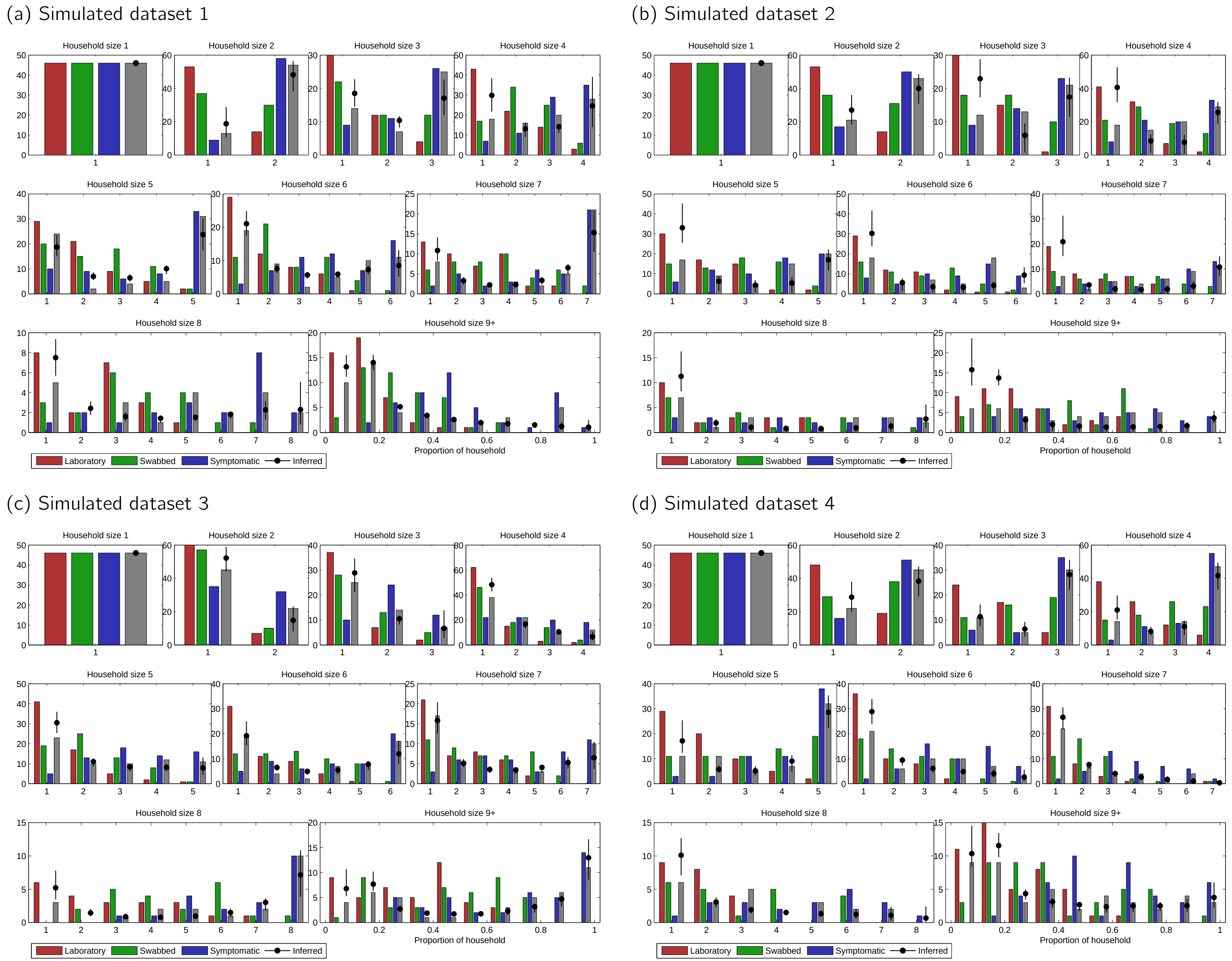

Figure S5: Histograms for simulated data. Values for cases obtained through simulation shown as grey bars.
